# A Review of Ecological Factors Associated with the Epidemiology of Wildlife Trypanosomiasis in the Luangwa and Zambezi Valley Ecosystems of Zambia

**DOI:** 10.1155/2012/372523

**Published:** 2012-05-27

**Authors:** Hetron Mweemba Munang'andu, Victor Siamudaala, Musso Munyeme, King Shimumbo Nalubamba

**Affiliations:** ^1^Section of Aquatic Medicine and Nutrition, Department of Basic Sciences and Aquatic Medicine, Norwegian School of Veterinary Sciences, Ullevalsveien 72, P.O. Box 8146 Dep, 0033 Oslo, Norway; ^2^Kavango Zambezi Transfrontier Conservation Area Secretariat, Kasane 821, Gaborone, Botswana; ^3^Department of Disease Control, School of Veterinary Medicine, University of Zambia, P.O. Box 32379, Lusaka 10101, Zambia; ^4^Department of Clinical Studies, School of Veterinary Medicine, University of Zambia, P.O. Box 32379, Lusaka 10101, Zambia

## Abstract

Trypanosomiasis has been endemic in wildlife in Zambia for more than a century. The disease has been associated with neurological disorders in humans. Current conservation strategies by the Zambian government of turning all game reserves into state-protected National Parks (NPs) and game management areas (GMAs) have led to the expansion of the wildlife and tsetse population in the Luangwa and Zambezi valley ecosystem. This ecological niche lies in the common tsetse fly belt that harbors the highest tsetse population density in Southern Africa. Ecological factors such as climate, vegetation and rainfall found in this niche allow for a favorable interplay between wild reservoir hosts and vector tsetse flies. These ecological factors that influence the survival of a wide range of wildlife species provide adequate habitat for tsetse flies thereby supporting the coexistence of disease reservoir hosts and vector tsetse flies leading to prolonged persistence of trypanosomiasis in the area. On the other hand, increase in anthropogenic activities poses a significant threat of reducing the tsetse and wildlife habitat in the area. Herein, we demonstrate that while conservation of wildlife and biodiversity is an important preservation strategy of natural resources, it could serve as a long-term reservoir of wildlife trypanosomiasis.

## 1. Introduction

Trypanosomiasis has been endemic in Zambia for a long time. The great African rinderpest pandemic which wiped out most of the ungulate population in the late 1880s contributed to the decline of the wildlife and tsetse distribution in Zambia [1, 2]. As a result, eastern Zambia was largely free of tsetse making cattle rearing virtually possible in the Luangwa valley towards the end of the 19th century [3]. However, the quick repopulation of wildlife enhanced the increase of the tsetse population density in the Luangwa valley. This lead to wild game, mainly elephants, and tsetse to expand southward and eastward onto the plateau areas resulting into new outbreaks of bovine trypanosomiasis after the rinderpest epizootic [4, 5]. Government attributed this expansion to occurrence of trypanosomiasis outbreaks in cattle and decided to establish the Department of Game and Tsetse Control in 1942 [6] whose main task was to eliminate wildlife hosts of tsetse as a control strategy [7]. This resulted in public opposition and was abolished in 1960. In the 1950s game fences were introduced to prevent the expansion of the wildlife population followed by the subsequent reinvasion of tsetse in the reclaimed “tsetse-free” areas [8, 9]. By 1968, spraying using endosulfan was introduced while odor-baited targets were introduced in 1970 to reduce tsetse infested areas [8, 9]. Put together, game fences, bush burning, aerial spraying, and use of odor-baited targets were used to reduce the expansion of tsetse further away from the Luangwa and Zambezi valley ecosystem. These practices were confined to communal areas unlike the National Parks (NP) in the valleys where wildlife and tsetse are allowed to interact freely.

In the 1940s ownership of wildlife changed hands from tradition control to state control. This led to creation of wildlife estates which restricted traditional hunting and access to protected areas by local people. As a result, the Luangwa valley was turned into game reserves in 1938 which put in place regulations forbidding illegal hunting of wildlife. This led to increase in the wildlife population in the area despite continued poaching from local tribes that were dependent on wildlife as a source of revenue. By 1970, the Luangwa valley had over 100,000 elephants (*Loxodonta Africana*) [10], while the Black rhinoceros was in the order of 4,000–12,000 [11]. As law enforcement began to collapse, illegal hunting increased leading to drastic reduction of some species that led to annihilation of the Black rhinoceros (*Diceros bi cornis*) and significant reduction of the elephant population in subsequent years [12]. By 1972, the government turned the game reserves into National Parks (NP) with the view to reduce illegal hunting and improve conservation strategies in order to regain the lost wildlife population in the area. Although species like warthogs (*Phacochoerus aethiopicus*) are still high, the overall wildlife population has been reduced due to poaching that has continued to persist in the area. Despite some species decreasing, the conservation strategy of preserving wildlife in state protected areas has significantly contributed to sustenance of tsetse rendering the Luangwa and Zambezi valley ecosystems, which lay in the “common tsetse fly belt,” to have the highest tsetse population density in Southern Africa [13]. This area provides a unique ecological niche that allows for a favorable interplay between wildlife reservoir hosts and vector tsetse flies. This niche is supported by a favorable climate and vegetation that sustains a large biomass of wildlife. In 1978, the Zambian government turned all areas surrounding the NPs into game management areas (GMAs) allowing for coexistence of wildlife and human habitation in the interface areas. Ultimately, this led to the “collision of the expanding tsetse fly belt with the expanding human population” making trypanoomiasis to be a threat to livestock and humans in the interface areas [14]. This paper reviews the ecological factors linked to the epidemiology of trypanosomiasis in the Luangwa and Zambezi valley ecosystems supported by the interplay between wildlife reservoir hosts, vector tsetse flies, and humans living in the interface areas using existing data and previous studies carried out in the area.

## 2. Ecological Habitats

### 2.1. Luangwa Valley Ecosystem

**Figure 1:**
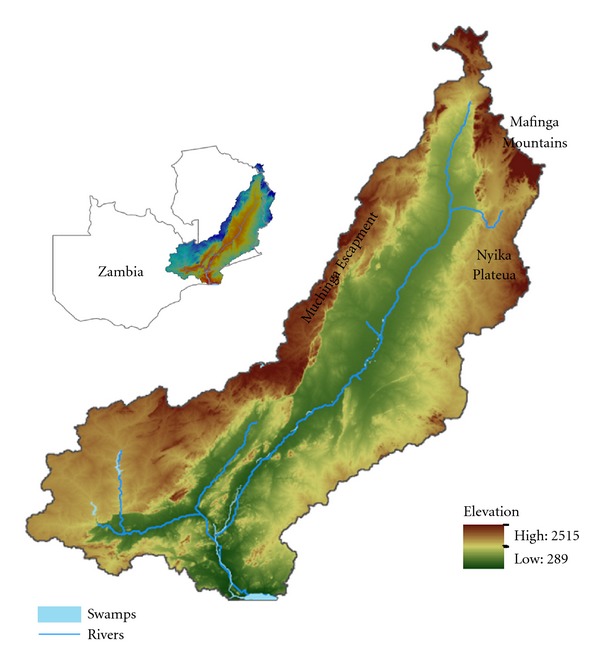
Shows the layout of the Luangwa valley. The Muchanga escapement is located on the western end of the valley at an altitude of 2,500 meters above sea level. The North is covered by the Mafinga mountains and Nyika Plateau while the valley flow lays below 500 meter above sea level. The Luangwa river is centered at the base of the valley floor.

**Figure 2:**
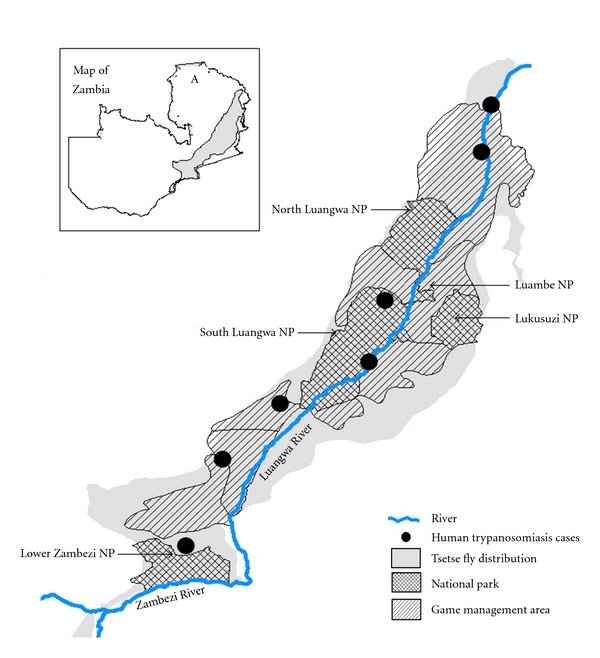
Shows the distribution of the tsetse infested areas overlapping the distribution of National Parks and Game Management Areas. Black dots show areas of clinical human trypanosomiasis cases, while insert shows the map of Zambia with A showing the study area.

Luangwa valley ecosystem is made of the Luangwa valley which stretches for a distance of 700 km with an average width of 200 km. The valley covers a total area of 63,000 km^2^ being part of the southern end of the Great Rift Valley that cuts across eastern Africa. It is covered by a biomass that sustains a vast range of wildlife and Glossina species. As shown in Figure 1, it is bordered by the Muchinga escapement, Mafinga Mountains, and Nyika plateau. The banks of the riverine are made of thick Miombo forests while the adjacent slopes are composed of Mopane woodlands. The valley floor is comprised of four NPs and six GMAs, namely, North Luangwa (4,636 km^2^), South Luangwa (9,050 km^2^), Luambe (247 km^2^), and Lukusuzi (2,729 km^2^) (Figure 2). Game management areas (GMAs) are buffer zones used for wildlife utilization mainly hunting unlike the NPs in which hunting is prohibited [15]. In addition, coexistence of wildlife and humans is permitted in GMAs and not NPs. The area has mean annual rainfall of 800 mm and an altitude of 500 m to 600 m. Daily ambient temperatures range from 32°C to 36°C, with mean minimum daily temperatures of 16°C to 23°C, respectively. The Luangwa valley is covered by Miombo woodlands. As pointed out by Lawton [16, 17], that Miombo and associated woodlands are the habitat of the tsetse fly, *Glossina morsitans*, the presence of which has a profound effect on the ecology and utilization of woodlands [18]. When there is wildlife in miombo, tsetse flies survive in large numbers. Hence, the combination of wildlife/miombo/tsetse fly is a natural ecosystem that sustains the persistence of trypanosomiasis for a long time. As shown in Figure 2, the Luangwa valley lies in an ecosystem that overlays NPs and GMAs with tsetse distribution being part of the famous “common fly belt” having the high tsetse infestation density that covers an estimated area of 322,000 km^2^ involving Malawi, Mozambique, Zimbabwe, and Zambia [13]. Robinson et al. [13] noted that in this common fly belt the highest tsetse densities are centered on the drainage systems of the Luangwa and Zambezi rivers. The most common species of tsetse in the area are *Glossina morsitans morsitans* Westwood and *Glossina pallidipes* Austen. Given the relative abundance of a diverse wildlife population and a high population density of *Glossina *species, this area renders the best ecological niche for trypanosomiasis transmission between tsetse and wild game.

### 2.2. Zambezi Valley Ecosystem

It encompasses the Lower Zambezi NP and the Luano, Chiawa, and Rufunsa GMAs. The NP covers an area of 4092 km^2^ and total GMA is estimated at 10361 km^2^. Similar to the Luangwa valley, the Lower Zambezi NP lays in a tsetse-infested area (Figure 2). The Lower Zambezi NP was established in 1983 which constitutes the valley floor bordered by the Zambezi river on the east. The riverine is covered by a thick forest which opens into the Mopane and Acacia woodlands. It is surrounded by the Zambezi escapement which makes the furthest end of the Great Rift Valley in the south. The mean annual rainfall is estimated at 700 m, while the mean maximum daily temperature varies between 32°C and 38°C. The mean minimum daily temperature fluctuates between 16°C and 24°C. The valley altitude varies from 350 m to 550 m. The common tsetse species found in the area are *Glossina morsitans morsitans* Westwood and *Glossina pallidipes* Austen. It is also located in the “common fly belt” with highest tsetse density being in the drainage systems of the Zambezi river [13]. 

## 3. Host Reservoir Behavior

### 3.1. Host Distribution

The relative abundance of wild game has a significant influence on the survival of tsetse. Some host species are widely distributed with lesser restrictive habitat requirements, attracting lesser poaching, less trophy hunting and having a much higher breeding potential, while some species are highly restrictive in their habitat requirements, highly poached, and attracting lucrative trophy hunting prices leading to large numbers being hunted every year. This makes some species to be ubiquitously distributed, while others are limited to selected ecological habitats best suited for their survival. For example, the warthog is a ubiquitously distributed species in the Luangwa and Zambezi valleys serving as one of the major sources of blood meals for tsetse while semiaquatic species like crocodiles and hippo are highly restrictive in their habitat requirements and as such are not commonly associated with trypanosomiasis. This would account for the reason why Okiwelu reported high proportion of warthog blood meal (62%) in a survey involving several species in the Luangwa valley [19]. Species such as the rhinoceros which have become extinct are no more a source of blood meal for tsetse and have been replaced by others. Hence the relative abundance of wild game makes a wide choice of blood meals for tsetse. Current conservation strategies aimed at reducing poaching in order to increase the wildlife population in these areas favor enrichment of the wildlife/miombo/tsetse ecosystems that sustains the persistence of trypanosomiasis. Given the wide host range of nocturnal species, the Luangwa and Zambezi valley ecosystems are likely to sustain trypanosomiasis for a long time.

### 3.2. Food Sources and Feeding Behavior

Differences in food sources among wild bovids tend to influence their contact with tsetse subsequently leading to varying degrees of exposure to trypanosomiasis infection. Kinghorn et al. [20] observed that waterbuck, bushbuck, eland, and kudu were the most heavily infected species among the bovids at Nawalia in the Luangwa valley (Table 1). They correlated their findings to the fact that these animals were usually found in thick cover from which they seldom emerged and as such they were more constantly exposed to tsetse bites than puku and wildebeest which were usually found in open country for the greater part of the day. These findings were supported by Keymer [21] Dillman and Townsend [22], and Rottcher [23] who observed a similar trend in the Luangwa valley after several decades (Table 1). In a more recent study, Anderson et al. [24] showed that waterbuck had the highest risk of trypanosomiasis infection followed by busbbuck and greater kudu. Hence, among the ruminants, browsers that mainly feed on tree leaves found in thickets where tsetse are mostly found are more prone to infection than the grazers found in open country, while semibrowsers that depend on both tree leaves and grazing are moderately susceptible. Anderson et al. [24] observed that habitat had a significant for species like the bushbuck that were sedentary in thickets and pointed out that such species had a higher risk of infection than nonsedentary species that moved widely covering large areas. Although this observation might be true for some habitats, studies have also shown that bulk grazers like the African buffalo that are lesser browsers are also prone to heavy infections. 

Okiwelu [25] showed that a close relationship between the feeding behavior of wild game and tsetse tends to influence disease transmission. Newberry et al. [26] observed a similar trend in the Luangwa valley that the daily feeding pattern of *Glossina morsitans morsitans* was highly correlated with host behavior. At two selected study areas near Mfuwe (Figure 1) elephants, rhinoceros, and hippopotamus entered the study areas at night and very early in the morning. Trypanosomes were highly detected from blood meals collected from these hosts in the morning collections only, while animals that were present throughout the day had trypanosomes in blood meals collected in the morning and evenings reflecting the diurnal feeding pattern of *Glossina spp*.

### 3.3. Diurnal versus Nocturnal Species

Okiwelu [25] observed that warthogs were among the most preferred hosts being active in the morning and late afternoon correlating with the *Glossina* flight activity which also had its peak early in the morning and late afternoon. Similarly Newberry et al. [26] and Clarke also reported that warthogs were a major host of *Glossina morsitans morsitans*. These findings reflect that diurnal species are more vulnerable to tsetse infestation, while the nocturnal species are lesser favorable hosts. Kinghorn et al. [27] examined 142 wild rats, 15 mice, one wild rabbit, one squirrel, one galago, and two genet all being nocturnal species and were found negative of trypanosomiasis. Although Okiwelu detected trypanosomes from blood meals of an aardvark which only feeds at night [25], studies carried out thus far indicate that nocturnal species are less favored hosts of tsetse subsequently being less infected by trypanosomes.

### 3.4. Seasonal Migrations

Seasonal variations in the movement of wild hosts has been reported to significantly influence the ecological behavior of *Glossina* species [26]. During the rain season, wild bovids are widely dispersed covering large areas within the park with water supplies being widely distributed in the Park. However, during the dry season as the water sources dry up, animals move to areas close to the river with tsetse densities increasing in area around the riverine. This trend of seasonal movement has a significant influence on the distribution of tsetse flies rendering the riverine area to be the most densely populated with tsetse populations [13].

## 4. Vectoral Behavior

### 4.1. Host Abundance

Robinson [28] pointed out that the principal factors that influence tsetse populations are host availability, climate, and vegetation. Host distribution and seasonal movement have a significant influence on the distribution of tsetse within an ecological habitat [29, 30]. Rainfall has been known to indirectly affect infection rates by significantly altering the relative abundance of wild game. In the rain season most bovids and suids are evenly distributed as watering points are widely distributed due to retention of water in various water reservoirs. As these watering points dry out in the dry season, wild game depend on the main rivers for water. Similarly the seasonal distribution of tsetse varies in correlation with the relative distribution of the wild hosts in each ecological Zone. In the dry season, the riverine vegetation which serves as the main source of green leaves for browsing species such as the kudu, waterbuck, and bushbuck also serves as the main resting places for tsetse as they keep away from the effect of excess heat. Animals are bitten by tsetse as they come for water to the main river. Hence seasonal movement of wild game influences local dispersal of tsetse within an ecological zone.

### 4.2. Climate and Relative Humidity

Temperature has a significant influence on the reproductive behavior and feeding habits of tsetse. Okiwelu [25] observed that favorable conditions increase the longevity of individual flies, enabling many flies to become infected and infection to mature. Significant correlations have been established between the mean monthly trypanosome infection rate and mean monthly temperature [25, 31]. Studies carried out in the Luangwa valley have shown that age structure of tsetse populations is largely dependent on temperature and relative humidity [26, 32–35]. Tsetse birth rate is generally low in the cold season when the pupal and interlaval periods are at their maximum [31, 35, 36]. Kinghorn carried out comparative studies to determine the impact of temperature on the life cycle of tsetse by comparing the hatchability at Nawalia in the Luangwa valley to that of the upland areas at high altitude. They observed that high altitude areas had long periods of low temperatures leading to the majority of the flies failing to emerge from puparia and many of those that did were malformed and quickly died. The hatchability in the valley was high and they attributed this to the short periods of low temperatures unlike the high altitude areas that had longer periods of low temperatures. Most flies caught from the Luangwa valley were infective by having infectious trypanosomes while those from the upland areas were less infective. They also compared the infectivity of flies caught from the upland plateau areas and those of the Luangwa valley during the hot season. They observed that flies from the Luangwa valley were three times more infective than the ones caught from the upland plateau implying that the ecosystem in the valley was more favorable to the breeding of tsetse than the plateau. This accounts for reasons why the disease has persisted in the Luangwa valley for over a century, mainly because of the favorable climatic conditions that favor the survival of tsetse flies and wildlife reservoirs.

### 4.3. Feeding and Resting Behavior

The nocturnal resting and feeding behavior of tsetse flies has been reported by Robinson [28] in the Luangwa valley and by R. D. Pilson and B. M. Pilson [37] in the Zambezi valley, while the diurnal feeding pattern in other places outside the Luangwa and Zambezi valley ecosystems has been reported by other scientist [25, 38]. R. D. Pilson and B. M. Pilson [37] observed that feeding was confined to temperatures between 18°C and 32°C with a median of 25°C in the Zambezi valley. Similar observations were made by Woolhouse et al. in the Luangwa valley [39]. Studies have shown that the feeding activity in the Luangwa and Zambezi valleys follow a diurnal pattern with Glossina species feeding more in the morning when ambient temperature rises above 18°C declining as the temperatures increase in the late part of the morning. Feeding resumes as the temperatures decline in late afternoon. This feeding regime makes Glossina species to have a diurnal feeding pattern. This behavior befits the resting behavior and feeding pattern of most bovids and suids that make the large source of food for tsetse suggesting that the feeding and resting behavior of both vector and hosts species has been synchronized to render an optimal transmission pattern of trypanosomes in the Zambezi and Luangwa valleys. Generally long periods of low temperatures tend to depress feeding rendering the cold season and extreme hot season not conducive for the survival of *Glossina* species. This makes the climate in the Luangwa and Zambezi valley to be more favorable than the upland areas.

### 4.4. Host Preference

In Zambia, host preference of *Glossina* species has been extensively studied by analyzing blood meal contents from mouth parts of tsetse by different scientists [25, 40]. As shown in Table 3, wild suids and bovids are the major sources of food for most tsetse species. Studies carried out in Luangwa and Zambezi valley ecosystems have shown that several factors influence the choice of host species as a source of bloodmeal for tsetse. These factors include the relative abundance of wild hosts, seasonal and daily movement of the host, feeding habits of the host, and the nocturnal or diurnal behavior of the host. However, tsetse easily adapts and will readily turn to other wildlife species in situations where the preferred species is not available. Newberry et al. [26] observed a switch in the choice of hosts in the Mfuwe area of the Luangwa valley where a small herd of buffaloes that provided the main meal for tsetse was replaced by warthogs when the buffalo herd was reduced due to predation by lions. The easy way with which tsetse switch from one host species to the other suggests that as long as there are alternative species serving as a source of food the reduction or extinction of one host species may not adversely affect the long-term survival of tsetse. For example, the extinction of the rhinoceros in the South Luangwa NP may not have caused a significant decrease in the survival of Glossina species given the high presence of alternative host species such as warthogs that have been thriving in large numbers in the same area. Hence, a wide host range is proponent to long-term survival of tsetse populations in an ecological habitat that has to support sufficient interplay between wildlife hosts and vector species. Conservation strategies put in place by the Zambian government aimed at reducing poaching to insignificant levels, restocking of extinct species such as the rhinoceros in North Luangwa NP, protecting endangered species, and control of wildlife diseases have led to the increase in the relative abundance of wild game thereby increasing the longevity of tsetse populations in the Luangwa and Zambezi valley ecosystems.

## 5. Vector and Reservoir Competence

### 5.1. Intrinsic Factors

As pointed out by Hardy [41], Woodrings et al. [42], and Goddard [43], vector competence refers to the intrinsic permissiveness of arthropods for infection, replication, and onward transmission of the pathogen to naïve hosts. Several studies based on dissecting tsetse to determine trypanosome infection rates in the mouth parts, midgut, and other organs of tsetse aimed at determining the infection rates and replication capacity of trypanosomes in Glossina species coupled with transmission to laboratory animals and wildlife hosts have been carried out by different scientists in Zambia [25, 44–46]. Intrinsic factors that influence the transmissibility of trypanosomes in tsetse include mature midgut infections [47], fly species [48], fly sex [49], and trypanosome genotype [50]. These intrinsic factors could account for the varying infection rates among tsetse observed from studies carried out in Zambia [25, 44–46]. Moloo and Gooding [51] showed that the vectorial competence of *Glossina morsitans centralis* originating from Zambia was comparable to that of Tanzania for *T*.* vivax* and *T*. *congolense* and was superior for *T*. *brucei*. This kind of vector competence is favorable for the longevity of sustaining a viable transmission cycle between wildlife reservoirs and tsetse in such a closed ecosystem. However, it is important to point out that data on vector competence is limited to the type of diagnostic tests used to determine infection rates in tsetse. Various techniques have been used for vector competence studies ranging from basic microscopy, laboratory animal inoculation, human-resistant-associated (SRA) gene to detect *T.b. rhodesiense*, molecular biology tools like PCR and genetic characterization using Minisatellite analysis. It is likely that using a combination of these tools will increase our understanding of the evolutionary aspects and competence of different vectors in the transmission of different trypanosomes.

### 5.2. Extrinsic Factors

complementary to intrinsic factors are the extrinsic factors which also play an important role in the vector competence of tsetse in transmitting trypanosomes from one host to the other. Extrinsic factors include climate and the relative abundance of wild hosts and their ability to carry trypanosomes in their blood for a long time. Temperature has been shown to influence the maturation of trypanosomes into infective forms within tsetse while influencing the growth pattern of tsetse. The better the climatic condition, the larger the number of adult flies and the more the number of flies with infective tsetse flies. Kinghorn et al. [27] reported an infection rate that was fives time higher in the Luangwa valley than the upland areas from experimentally bred tsetse flies and an infection rate that was 2.5 times higher from naturally infected tsetse in the Luangwa valley than the upland areas. At Kakumbi in the Luangwa valley, Woolhouse et al. [46] estimated the overall *per capita* rate of infection at 0.37% of the flies acquiring new infections per day which is much higher than infection rates reported elsewhere [46]. Hence in a balanced ecosystem that allows for adequate interplay between wildlife hosts and tsetse as observed in the Luangwa valley ecosystem, vector competence compliments reservoir competence.

### 5.3. Reservoir Competence

This reflects the ability of animal hosts to maintain infectious trypanosomes transmissible to other naïve hosts. Dillman and Townstsend [52], Kinghorn and York [53], and Rickman et al. [54] showed infectivity of trypanosomes collected from asymptomatic warthogs, waterbuck, impala, hartebeest, bushbuck, giraffe, and kudu when infected in mice and monkeys suggesting that trypanosomes were adaptive to a wide host range.  Table 2 shows different *trypanosoma* species detected from different wildlife species in the Luangwa and Zambezi valleys. Recently, Anderson et al. [24] detected *T. b. rhodesiense* in the African Buffalo and *T. Brucei. s.l.* in leopard for the first time showing that the reservoir community for trypanosomes is even wider than previously thought. Infection studies carried out using *T. vivax*, *T. brucei*, and *T. congolense* on nonimmune wild and domestic animals bred in captivity in a tsetse-free area in Kenya showed that the incubation period in wild game was longer than in domestic animals and that wildlife developed low parasitaemia with low anaemia compared to domestic animals that had high parasitaemia and anaemia leading to chronic infections [55]. Similarly field observations from game shot in the Luangwa valley showed low parasitaemia and low infection rates [20–22, 56]. Based on these observations, Dillman and Townsend [52] concluded that several wildlife species live in equilibrium with trypanosomes carrying the hemoparasites without expressing clinical signs by reaching a degree of trypanotolerance in the Luangwa valley. Thus far, clinical trypanosomiasis in wild game in Zambia has only been reported from a lion obtained from Chichele springs in the Luangwa valley [57]. The ability of wild hosts to maintain viable trypanosomes is vital for continuous transmission of the disease between wildlife and tsetse. Immunological responses that permit the survival of trypanosomes in wild game have been reviewed by Mulla and Rickman [55]. Generally, they observed that the incubation period in wildlife was longer than in domestic animals and that wild game developed low parasitaemia than domestic animals with reservoir ability to maintain trypanosomes for a long time.

## 6. Human Trypanosomiasis

The first report of *Trypanosoma brucei rhodesence* in humans dates as far back as 1908 when the first case of sleeping sickness was reported from a person living in the Luangwa valley [2]. Although the zoonotic *trypanosoma* has been isolated by different scientists from different wildlife species in the area (Table 2), its prevalence in humans is highly variable. Rickman [2, 58] reported of human infection rates of 12.3% in the Petauke area and Buyst reported of infection rates of 16.5% in Isoka [2]. Recently Anderson et al. [24] reported of a low prevalence of 0.5%  (*n* = 418) of *T.b. rhodesiense* in wildlife in the Luangwa valley indicating that maintaining biodiversity in the Luangwa valley could have influenced the limited emergence of this parasite in the Luangwa valley ecosystem. This observation contradicts the common perception that spillover from wildlife is a risk factor for humans living in GMAs as earlier indicated by Buyst [2] who observed an increase in tsetse populations around the villages during the dry season when wildlife had retreated to the riverine in the NPs followed by a more wide spread distribution away from villages in the rain season when wildlife were evenly distributed. These observations led Buyst [2] to conclude that tsetse switch to humans as an alternative source of blood meal when wild animals were scarce during the dry season and that they resorted back to wildlife during the rainy season when wild animals were evenly distributed in GMAs and NPs. These assertions have been supported by observations made by other scientists as shown in Table 3 that humans living in GMAs also serve as sources of blood-meal for tsetse. As shown in Figure 2, clinical cases of human trypanosomiasis have been reported in GMAs although asymptomatic cases are also common [59]. However, to fully understand the role of humans living in GMAs on the epidemiology of trypanosomiasis, there is need for further investigations.

## 7. Anthropogenic Activities

Anthropogenic activities in the Luangwa and Zambezi valleys adversely affecting the ecosystem include illegal hunting, deforestation, charcoal burning, cultivation, and pastoralist. Among these, illegal hunting is the most important. Lewis et al. [60] estimated the human population density in the valley floor at less than 10 persons per km^2^ except for the mid-Luangwa valley area that had a higher density above 30 persons per km^2^ due to increased tourism and pastoralists. Timberlake and Chidumayo [61] pointed out that in miombo woodlands, areas having population densities above 10 persons per km^2^ are threatened by anthropogenic pressure due to increased demand for natural resources. Hence, the low human population density in the valleys (<10 persons/km^2^) would account for reasons why the tsetse population density is high in this area because of less competition for natural resources between humans and wildlife. On contrast, it has been shown that human dependence on wildlife is high in the Luangwa valley [15]. This has for a long time been exacerbated by the nonpastoralist tribes of northern Zambia that relied on illegal hunting as a source of livelihood. By 1999, it was estimated that 40–60% of the valley residents were unable to produce enough food and relied on illegal hunting as an alternative source of income. Lewis et al. [15] have estimated that more than 3,000 hooved animals were killed by illegal hunters in the Luangwa valley every year. As a result, wild animals tend to retreat into the NPs where they are protected by patrolling game rangers avoiding the GMAs where the hunting pressure is high. This accounts for reasons why the high population density of both wildlife and tsetse is higher in the NPs rendering this area to be part of the common tsetse fly belt having the highest tsetse population density in Southern Africa.

**Figure 3:**
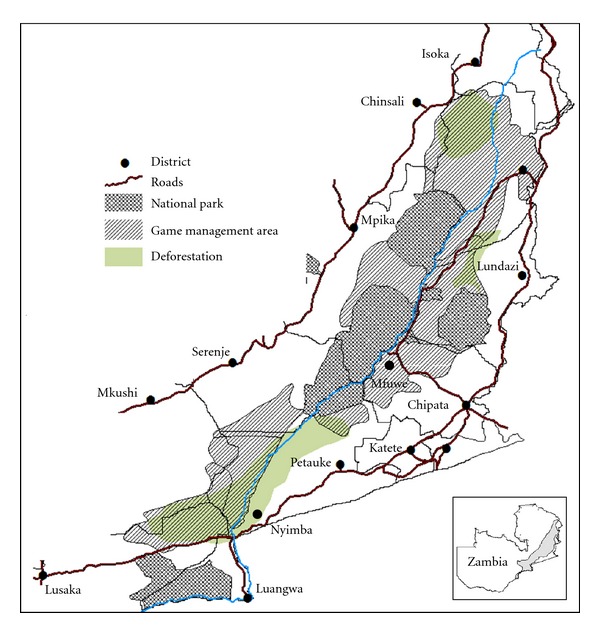
Shows the districts surrounding the National Parks and Game Management areas (GMAs) and deforestation in the GMAs. Inset shows the map of Zambia and study area.

Deforestation due to charcoal extraction and clearing of land for cultivation and livestock production are contributing to loss of habitat for wildlife and tsetse in the GMAs. More than 31,000 tons of charcoal is extracted annually in the Nyimba and Petauke GMAs (Figure 3). A similar trend has been reported in the Chinsali GMAs, while more than 21% of the forest cover in Lundazi has been cleared for crop cultivation [62]. As pointed out by Lawton [16], an ecosystem of human settlement/cultivation/pastoralism is opposite of the wildlife/miombo/tsetse ecosystem that naturally sustains the persistence of trypanosomiasis. Once miombo is cleared, tsetse and wildlife retreat into NPs due to loss of habitat in GMAs giving way to crop cultivation or pastoralism so long miombo woodlands do not regenerate for reinvasion of tsetse. Similarly, extensive burning of ground cover annually done by local residents to facilitate wild honey collection, hunting and crop cultivation contributes to loss of habitat although early burning is known to favor regeneration of woodlands which creates a favorable habitat for tsetse while late burning in the dry season creates open areas unfavorable for tsetse. To counteract these adverse anthropogenic effects, conservation strategies aimed at empowering local residents with alternative income generating activities are been explored [60]. More than 25,000 beehives have been established in the area while illegal hunters are encouraged to surrender their firearms and snares in exchange for alternative income sources [60]. Overall, positive attributes gained from these activities include increase in wildlife populations, even distribution of wildlife due to reduced hunting pressure and reduced in deforestation.

## 8. Conclusion

It is evident from this paper that trypanosomiasis can be maintained in a closed ecosystem for more than a century so long ecological factors that favor adequate interplay between tsetse and wildlife reservoir hosts are kept in balance in a suitable habitat. Although anthropogenic activities in the area seem to adversely affect the expansion of wildlife by shrinking the ecological habitat of wildlife and tsetse, in the NPs where human interference is low due to presence of patrolling game rangers and other law enforcement officers, there is high interaction between tsetse and wildlife rendering the Luangwa and Zambezi valley ecosystems to be one on the most tsetse densely populated areas in Southern Africa [66].  Table 4 summarizes some of the ecological and biological factors influencing the epidemiology of trypanosomiasis in the Luangwa and Zambezi valley ecosystems. It is eminent from the enlisted biological and ecological factors that persistence of wildlife trypanosomiasis in an ecosystem is dependent on interplay of several factors that include vector tsetse flies, wildlife, habitat, and conservation strategy. It is clearly demonstrated from the historical perspective of trypanosomiasis in the Luangwa and Zambezi valley ecosystems that the disease which was almost wiped out due to loss of wildlife during the rinderpest pandemic, reemerged and has been maintained through shift in policies that have evolved from traditional ownership of wildlife to state protected properties by introducing game reserves during the precolonial era to establishment of NPs and GMAs in the 1970s until the current conservation strategies that are aimed at increasing the existing wildlife population and restocking formerly annihilated species such as the Black rhinoceros. Put together, these efforts demonstrate that while conservation of biodiversity is an important preservation strategy of natural resources, it could also serve as long-term reservoir of trypanosomiasis.

## Figures and Tables

**Table 1:** Wildlife species tested for the presence of trypanosomiasis.

Wildlife species	Year/period reference					
	1911-1912 [20]	1962 [21]	1971–1974 [22]	1977 [23]	2007	Total
Baboon (*Papio cynocephalus*)			0/20	3/4		3/24
Bat (*Nycteris species*)			2/2			2/2
Black rhinoceros (*Diceros bi cornis*)	0/1					0/1
Buffalo (*Syncerus caffer*)		0/4	2/19	14/32	7/65	23/120
Bushbuck (*Tragelaphus scriptus*)	6/9	3/6	14/23		11/28	34/66
Bushpigs (*Potamochoerus porcus*)	0/1					0/1
Cane rat (*Thryonomys *species)			0/1			0/1
Civet (*Viverra civetta*)			1/6			1/6
Crocodile (*Crocodylus niloticus*)			0/1		0/5	0/6
Duiker (*Sylvicapra grimmia*)			3/7	3/3	0/2	6/12
Eland (*Taurotragus oryx*)			2/3	0/4	0/1	2/7
Elephant (*Loxodonta Africana*)			2/20	11/23	0/7	13/50
Genet (*Genetta genetta*)			0/6			0/6
Giraffe (*Giraffa camelopardalis*)			1/1		0/1	1/2
Grysbok (*Raphicerus species*)			0/5		0/4	0/9
Hare (*Hepus capensis*)			0/10	0/14		0/24
Hartebeest (*Alcelaphus lichtensteini*)	1/6	0/1		2/3	0/4	3/14
Hippo (*Hippopotamus amphibious*)	0/1		4/250	14/30	2/29	20/310
Hyena (*Crocuta crocuta*)			4/7	2/2	0/7	6/16
Impala (*Aepyceros melampus*)	2/29		1/23	2/27	4/47	9/126
Jackal (*Canis mesomelas*)			0/1	2/15		2/16
Kudu (*Tragelaphus strepsiceros*)	4/7	1/1	11/13	5/86	8/20	29/127
Leopard (*Panthera pardus*)			0/2	1/1	2/14	3/17
Lion (*Panthera leo*)			6/6	1/2	6/14	13/22
Mongoose (*Herpestes sanguineus*)			0/2			0/2
Monkey vervet (*Cercopithecus species*)			0/18		0/1	0/19
Puku (*Kobus vardoni*)	1/10	2/5	1/24	12/40	3/57	19/136
Porcupine (*Hystrix galeata*)			0/1			0/1
Reedbuck (*Redunca redunca*)					1/1	1/1
Rhinoceros (*Diceros bicornis*)	0/1		0/5	0/1		0/6
Roan antelope (*Hippotragus equinus*)	1/8		2/11	2/10	0/5	5/34
Serval (*Felis serval*)			0/2			0/2
Warthog (*Phacochoerus aethiopicus*)	1/9	0/3	6/24		7/56	16/92
Waterbuck (*kobus ellipsiprymnus*)	17/28	4/7	16/20		6/10	43/60
Wild cats (*Felis lybica*)			0/1			0/1
Wild dog (*Lycaon pictus*)			0/2	1/1	0/3	1/6
Wildebeest (*Connochaetes taurinus*)	0/2	0/3	1/5	6/37	1/10	8/57
Zebra (*Equus burchelli*)	0/5	0/6	0/5		0/27	0/43

Total examined	33/119	10/43	79/546	64/10	58/418	174/1232

**Table 2:** *Trypanosoma* species detected from wildlife in the Luangwa and Zambezi valleys.

*Trypanosoma* species	Wild animal hosts	References
*Trypanosoma vivax*	Bushbuck (*Tragelaphus scriptus*)	[20–22]
	Buffalo (*Syncerus caffer*)	[22–24]
	Common duiker (*Sylvicapra grimmia*)	[22]
	Eland (*Taurotragus oryx*)	[22]
	Hippo (*Hippopotamus amphibious*)	[24]
	Kudu (*Tragelaphus strepsiceros*)	[20, 22–24]
	Puku (*Kobus vardoni*)	[22]
	Roan antelope (*Hippotraggus equinus*)	[20, 24]
	Reedbuck (*Redunca redunca*)	[24]
	Waterbuck (*Kobus ellipsiprymnus*)	[20–22, 24]
	Warthog (*Phacochoerus aethiopicus*)	[24]
	Wild dog (*Lycaon pictus*)	[23]
	Wildebeest (*Connochaetes taurinus*)	[22]

*Trypanosoma congolense*	Baboon (*Papio cynocephalus*)	[23]
	Buffalo (*Syncerus caffer*)	[22–24]
	Bushbuck (*Tragelaphus scriptus*)	[20–24]
	Civet (*Viverra civetta*)	[22]
	Common duicker (*Sylvicapra grimmia*)	[22]
	Eland (*Taurotragus oryx*)	[22]
	Elephant (*Loxodonta Africana*)	[22, 23]
	Hyaena (*Crocuta crocuta*)	[22, 23]
	Hippo (*Hippopotamus amphibious*)	[24]
	Impala (*Aepyceros melampus*)	[20, 22, 24]
	Kudu (*Tragelaphus strepsiceros*)	[20, 22, 24]
	Puku (*Kobus vardoni*)	[23, 24]
	Lion (*Panthera leo*)	[22–24]
	Roan antelope (*Hippotraggus equinus*)	[20, 22, 23]
	Waterbuck (*Kobus ellipsiprymnus*)	[20–22]
	Wild dog (*Lycaon pictus*)	[23]
	Warthog (*Phacochoerus aethiopicus*)	[22, 24]
	Wildebeest (*Connochaetes taurinus*)	[24]

*Trypanosoma brucei*	Buffalo (*Syncerus caffer*)	[24]
	Bushbuck (*Tragelaphus scriptus*)	[20, 22, 24]
	Giraffe (*Giraffa camelopardalis*)	[22]
	Hayna (*Crocuta crocuta*)	[22, 23]
	Hartebeest (*Alcelaphus lichtensteini*)	[20]
	Hippopotamus (*Hippopotamus amphibious*)	[22, 24]
	Impala (*Aepyceros melampus*)	[20, 23, 24, 63]
	Lion (*Panthera leo*)	[22–24]
	Puku (*Kobus vardoni*)	[24]
	Warthog (*Phacochoerus aethiopicus*)	[20, 22, 24]
	Waterbuck (*Hippotraggus equinus*)	[20, 22, 24]
	Wildebeest (*Connochaetes taurinus*)	[24]
	Wild dog (*Lycaon pictus*)	[23]
	Zebra (*Equus burchelli*)	[63]

**Table 3:** Animal blood meals detected from *Glossina spp*. in Zambia.

Host	References
Aardvark (*Orycteropus afer*)	[19]
Baboon (*Popio cynocephalus*)	[19]
Bird (Unclassified)	[19, 64]
Buffalo (*Syncerus caffer*)	[19, 64, 65]
Bushbuck (*Tragelaphus scriptus*)	[19, 64]
Bushpig (*Potamochoerus larvatus*)	[19, 64]
Carnivore (unclassified)	[19]
Duicker (*Sylvicapra grimmia*)	[19, 64]
Eland (*Taurotragus oryx*)	[64]
Elephant (*Loxodonta Africana*)	[19, 64, 65]
Goats (unclassified)	[64, 65]
Hippo (*Hippopotamus amphibious*)	[19, 64]
Kudu (*Tragelaphus strepsiceros*)	[19, 64, 65]
Man (*Homo sapiens sapiens*)	[19, 64, 65]
Monkey (*Cercopithecus species*)	[19, 64]
Porcupine (*Hystrix africaeaustralis*)	[19]
Primates (unclassified)	[19, 64]
Puku (*Kobus vardoni*)	[19]
Rhinoceros (*Diceros bicornis*)	[64]
Roan antelope (*Hippotraggus equinus*)	[19, 64]
Rodents (unclassified)	[19]
Reptile (unclassified)	[19, 64]
Waterbuck (*Hippotraggus equinus*)	[19, 64]
Warthog (*Phacochoerus aethiopicus*)	[19, 64, 65]

**Table 4:** Ecological and biological factors influencing the epidemiology trypanosomiasis.

Variable	Ecological/biological factor	Influence/effect
	Vector species	*Glossina morsitans morsitans*, *Glossina brevipalpis *
Vector	Intrinsic factors	Increase in vector competence Favorable for survival and reproduction of trypanosomes
	Extrinsic factors	Favorable environment for completion of tsetse life cycle Supportive climate in the valley for survival of vector species

	Relative abundance	Wide choice of feed for vector species Easy choice of alternative feed sources for the vectors
Wildlife	Wide host species	Easy choice of alternative blood-meal options for vectors Extinct species are easily replaced
	Trypanotolerance	Long-term carriers of viable trypanosomes to vectors Increased host competence-tolerance of high infection rates
	Feeding behavior	Nocturnal species synchronized with vector feeding behaviors Diurnal species less favorable by vector species

		Valley temperature favorable for survival of host species
	Climate	Short duration of cold months Rain season favors wide dispersal of host reservoir
Habitat		Valley temperature is favorable for breeding of vector species
	Vegetation	Plant species source feed to wildlife host reservoir Ideal for hibernation of tsetse flies away from the heat
	Riverine	Source of water for host species during dry season Favorable vegetation for survival and hibernation of tsetse flies

	NP	Reduced poaching—increase in host reservoir population High tsetse population density—No eradication programs
Conservation		Expansion of interface—human encroachment
	GMA	Low wildlife population—poaching, human/wildlife conflicts Livestock/humans become alternative sources of blood meal
		Increased risk of human exposure to trypanosomiasis
